# Food claims and nutrition facts of commercial infant foods

**DOI:** 10.1371/journal.pone.0191982

**Published:** 2018-02-28

**Authors:** Yu-Chin Koo, Jung-Su Chang, Yi Chun Chen

**Affiliations:** School of Nutrition and Health Science, Taipei Medical University, Taipei, Taiwan; University of Miami, UNITED STATES

## Abstract

Composition claim, nutrition claim and health claim are often found on the commercial complementary food packaging. The introduction of complementary foods (CFs) to infants is a turning point in the development of their eating behavior, and their commercial use for Taiwanese infants is growing. In Taiwan, lots of the advertisements for CFs employed health or nutrition claims to promote the products, but the actual nutritional content of these CFs is not clear. The aim of this study was to compare the food claims of commercial complementary food products with their actual nutrition facts. A sample of 363 commercial CFs was collected from websites, local supermarkets, and other food stores, and their nutrition-related claims were classified into composition, nutrition, and health categories. Although the World Health Organization recommends that infants should be exclusively breastfed for the first 6 months, 48.2% of the commercial CFs were targeted at infants younger than 6 months. Therefore, marketing regulations should be implemented to curb early weaning as a result of products targeted at infants younger than 6 months. More than 50% of Taiwanese commercial CFs have high sugar content and more than 20% were high in sodium. Products with health claims, such as “provides good nutrition to children” or “improves appetite,” have higher sodium or sugar content than do those without such claims. Moreover, products with calcium or iron content claims did not contain more calcium or iron than products without such claims. Additionally, a significantly greater proportion of the products with “no added sugar” claims were classified as having high sugar content as compared to those without such claims. Parents cannot choose the healthiest food products for their children by simply focusing on food claims. Government should regulate the labeling of nutrition facts and food claims for foods targeted at infants younger than 12 months.

## Introduction

The introduction of complementary food (CF) to infants is a turning point in the development of their eating behavior. The World Health Organization (WHO) recommends that infants should be exclusively breastfed for the first 6 months and then start receiving CFs to provide critical nutrients (e.g., iron and zinc) for their development [1–3]. Without a nutrition balanced diet, an infant might develop a nutritional deficiency. The general rule for introducing CFs to infants is to provide an assortment of flavors and textures so that they can learn to accept a variety of foods [4]. Moreover, infants’ sugar and salt intake should be limited so that they develop healthy eating habits and avoid chronic diseases later in life [5,6].

Commercial CFs are convenient to include in an infant’s diet. There are many different commercial CFs available—infant cereals, fruit purees, vegetable purees, meat purees, and infant cookies. An online survey of U.S. parents found that most select their children’s food and drinks according to the ingredients or claims on the product packaging; parents prefer drinks with low-calorie and natural claims [7]. A study discovered that parents perceived products with health or nutrition claims to be more nutritious for their children [8]. However, a study in the United States showed that more than 70% of children’s foods with specific nutrient content claims were high in sugar, sodium, or fat [9]. A study in Canada also found that more than 60% of commercial foods for infants and toddlers were high in sodium or sugar [10]. To prevent these food claims from misleading parents to purchase foods that are unhealthy for their children, the Codex Alimentarius Commission [11] stipulates that “nutrition and health claims shall not be permitted for foods for infants and young children except where specifically provided for in relevant Codex standards or national legislation.” Nevertheless, the rules do not apply in some countries. For example, no special rules exist in Taiwan regarding the health and nutrition claims of food for infants and young children.

Recently, the use of commercial CFs for Taiwanese infants has been increasing [12]. In popular Taiwanese pregnancy and early parenting magazines, 81% of the advertisements for CFs employed health or nutrition claims to promote the products, but the actual nutritional content of these CFs is not clear [13]. Therefore, this study investigated the food claims compared with actual nutrition facts for commercial CF products. The results may guide nutrition educators and future revisions of relevant national laws.

## Material and methods

### Data collection

Content analysis was used to investigate the packaging information on commercial CF products. In accordance with methods used in previous studies [14–16], the samples were collected from websites, local supermarkets, and other food stores. We identified food products for purchase from the sections labeled “baby” or “infant” foods.

Infant foods produced by the 16 main infant food manufacturers in Taiwan were sought during the period of September 2015 to February 2016. The final sample consisted of 363 commercial CFs. Packaging information on each product was obtained from manufacturer websites and by viewing products in the store. All the information on the food packaging was photocopied for reference and stored for subsequent content analysis. No approval was required for collecting these data because they were publicly available.

All of the samples were semisolid or solid infant foods; drinks, soup stock, and milk products were excluded. Infant drinks or soup is seldom used in Taiwan. Milk products, such as infant or growing-up formula, are ruled under different pieces of legislation. Products included simple purees or blended foods (soft, wet, and ready-made food); infant cereals (powders that mix with breast milk or water); and infant cookies (e.g., biscuits, puffs, and teething cookies). Products with a single food ingredient, typically a starchy food, fruit, vegetable, or meat, are referred to as simple pureed foods; blended foods are products with two or more food ingredients (e.g., purees mixing multiple foods, or porridge).

### Coding process and categories

During the sample selection and photocopying process, common themes were noted and recorded. The first author used these themes to develop a coding form, which was used to train two other researchers who subsequently pretested it using a sample of ads. The pretest verified that the coding form included the distinct categories required to classify the contents of these ads, although several new categories were added as necessary during the coding of the final sample. This process also verified that the categories were understood and used in the same way by the three researchers. Some minor modifications to the form were made to improve clarity. Subsequently, all of the photocopied ads were coded separately by the two researchers, who consulted the first author over challenging coding decisions.

As Table 1 shows, the information was coded according to the following variables: basic information, product category, recommended age, nutrition facts, high sugar content, high sodium content, and food claims. First, the packaging information of the products was reviewed, classified, and coded. Second, the recommended age and nutrition-related message of each product were coded. The recommended age by manufacturers was grouped into the following categories: Stage 1 (4–6 months), Stage 2 (7–9 months), and Stage 3 (10–12 months). These age categories are commonly used on CF food packages in Taiwan.

**Table 1:** Content coding of packaging information.

Coding Variables	Coding Options
**Basic information**	Brand name
	Product name
	Number of product
**Product category**	Infant cereals, simple pureed foods, mixed foods, infant cookies
**Recommended age**	Stage 1, Stage 2, Stage 3
**Nutrition facts**	Calories, protein, total fat, statured fat, carbohydrate, sugar, and sodium content. We also recorded calcium and iron content if displayed on the packaging.
**High sodium content**	Yes/no, according to nutrition facts
**High sugar content**	Yes/no, according to nutrition facts
**Composition claims**	No added preservatives, no added coloring agents, no added seasoning, organic food, dairy-free, no added condiments, natural, fresh, gluten-free, contains vegetables, no allergens, no food additives, non-GMO food, no maltodextrin or modified starch, whole grain, no added artificial flavor, contains meat
**Nutrition claims**	Contains calcium, contains iron, contains vitamin C, contains a host of nutrients, contains dietary fiber, contains multiple vitamins, no added sugar, no added salt, contains vitamin E, contains multiple minerals, contains vitamin A/β-carotene, contains vitamin B_1_, contains ω-3, low sodium, contains zinc, contains probiotics or prebiotics, contains protein or amino acids, contains vitamin B_2_, contains phospholipid, contains iodine, contains phosphorus, contains vitamin D, contains DHA, contains lactose, contains carbohydrate, contains magnesium, contains selenium, contains arachidonic acid
**Health claims**	Nutritionally balanced, provides good nutrition to children, improves appetite, suitable for picky eaters, supports healthy growth, improves growth, good for digestion and absorption, supports learning to chew, supports learning to hold, combats constipation, good for bones and teeth, good for enteric flora, good for the brain, good for the eyes, supports vision and skin health, good for defecation, good for thyroxine synthesis, good for red blood cell synthesis and preventing iron-deficiency anemia, good for metabolism, good for collagen synthesis

### Food claims and nutrition facts

Prior to coding, all nutrition-related text that mentioned composition, nutrition, or health was typed exactly as it appeared on the packaging. Then claims were classified into composition, nutrition, and health categories. Composition claims refer to overarching representations of food ingredients or food quality (e.g., “organic food”) or the exclusion of ingredient types (e.g., “gluten-free” or “no preservatives”) [17]. Nutrition claims refer to any representation that states or suggests that a food has, or excludes, a particular nutrient (e.g. “contains iron” or “no added salt”) [11,18]. Health claims refer to any representation that states or suggests that a relationship exists between a food (or a constituent of that food) and health, such as nutrient function claims (e.g., dietary fiber improves digestion). This also includes claims that consuming the food (or its constituents) influences the normal functions or biological activities of the body in the context of the wider diet (e.g., contributing to balanced nutrition or an improved appetite) [11].

The nutrition facts per 100g listed on food packaging were then coded. According to regulations on nutrition labeling in Taiwan, packaged food should be labeled with the calorie, carbohydrate, protein, total fat, sugar, sodium, saturated fat, and trans fat content. Some of the infant cereals and cookies listed calcium or iron content, and this was also recorded. The nutrition fact data for infant cereals were recorded after the powder was blended with water by following the preparation methods on the packaging. For example, three spoonfuls (90 g) of infant cereal powder was added to 210 mL of water for a Stage 1 product, and four spoonfuls (120 g) of cereal powder was added to 240 mL of water for a Stage 2 product. Then, the nutritional content of 100 g of infant cereal meal was recorded.

High sodium content and high sugar content were also coded. A relevant study classified infant and toddler food with less than 130 mg of sodium per 100 g of product as “acceptable,” which is the AI reference intake for children aged 1–3 years in Canada’s Food Guide [10]. The age categories in this study were all ≦1 year; therefore, foods with 130 mg sodium or more were considered to have high sodium content. According to WHO [6] recommendations, both adults and children should derive less than 10% of their daily calories from sugar. Therefore, foods with 10% or more calories derived from sugar were defined as having high sugar content.

### Intercoder reliability and statistical analysis

Kappa analysis was performed to assess the intercoder reliability between the two independent coders (0.41–0.60 = moderate; 0.61–0.80 = substantial; 0.81–1.00 = almost perfect) [19]. This analysis was performed using SPSS version 19.0. Intercoder reliability between the two researchers was pilot-tested using the original content analysis form. The final kappa analysis was calculated from final coding of all ads which were coded by both researchers. All the kappa statistics were higher than 0.80 and thus deemed acceptable.

Results are expressed as frequencies (n), percentages (%), or the mean ± SD. Pearson’s chi-square test was used to examine the associations between two variables (for example, between the food categories, food claims, and salt or sugar content for each age stage). The Kolmogorov–Smirnov test was used to test the normal distribution of all continuous variables. Statistical difference among the groups was evaluated using a one-way analysis of variance (ANOVA) test with the Scheffé or Kruskal–Wallis test. The Mann–Whitney U test with Bonferroni adjustment was applied to examine pairwise differences followed by significance using the Kruskal–Wallis test. Kruskal–Wallis test and The Mann–Whitney U test were used when the variables are not normally distributed. Statistical analyses were performed using SPSS version 19.0. A *p* value lower than 0.05 was considered significant.

## Results

A total of 363 CF products were considered. Most of the products displayed a recommended age on the package label, although 1.1% (*n* = 4) did not display a recommended age and 34.7% (*n* = 126) did not display the sugar content. Therefore, the recommended age (*n* = 359) and sugar content (*n* = 237) were investigated only if they were displayed on the label.

### Distribution of different commercial CFs

Among all the CFs, 58.2% of the products were mixed foods (*n* = 211), 15.4% were simple pureed foods (*n* = 56), 13.2% were infant cereal (*n* = 48), and 13.2% were infant cookies (*n* = 48). In the CF product names, 27.1% of infant cereals (*n* = 13) and 42.9% of simple pureed foods (*n* = 24) included fruit in the name description (e.g., “Apple Puree”). Table 2 shows the distribution of CFs across the various food categories. Among all the CFs, 48.2% were recommended for Stage 1 infants, 25.9% for Stage 2 infants, and 25.9% for Stage 3 infants. Most infant cereals and simple puree foods were for Stage 1 infants, and more than 30% of the mixed foods were for Stage 1 and Stage 3 infants. Approximately 70% of infant cookies were for Stage 2 infants.

**Table 2:** Distribution of CFs across product categories ^1^.

	*N (%)*	Infant cereal	Puree food	Mixed food	Infant cookies	*p* value
**Total**	363 (100.0)	48 (100.0)	56 (100.0)	211 (100.0)	48 (100.0)	
**Stage**^2^						
** 1**	173 (48.2)	37 (84.1)	52 (92.8)	78 (36.9)	6 (12.5)	<0.001**
** 2**	93 (25.9)	7 (15.9)	1 (1.8)	51 (24.2)	34 (70.8)	
** 3**	93 (25.9)		3 (5.4)	82 (38.9)	8 (16.7)	
**Food claims**						
** Composition claim**	320 (88.2)	45 (93.8)	49 (87.5)	184 (87.2)	42 (87.5)	0.645
** Nutrition claim**	151 (41.6)	44 (91.7)	16 (28.6)	52 (24.6)	39 (81.3)	<0.001***
** Health claim**	178 (49.6)	41 (85.4)	13 (23.2)	107 (50.7)	21 (43.8)	<0.001***
**No added salt**	19 (5.2)	2 (4.2)	1 (1.8)	11 (5.2)	5 (10.4)	0.256
**No added seasoning**	78 (21.5)	0 (0.0)	13 (23.2)	65 (30.8)	0 (0.0)	<0.001****
**High sodium content**	85 (23.4)	0 (0.0)	1 (1.8)	55 (26.1)	29 (60.4)	<0.001***
**No added sugar**	50 (13.8)	24 (50.0)	11 (19.6)	8 (3.8)	7 (14.6)	<0.001***
**High sugar content**^3^	129 (54.4)	26 (68.4)	29 (70.7)	50 (41.0)	24 (66.7)	<0.001***

^1^ Data are presented as the number (percentage).

^2^ Only 359 products with stage data are accounted for in this table.

^3^ Only 237 products with sugar content data are accounted for in this table.

*p < 0.05;

**p < 0.01;

***p < 0.001 by chi-square test.

Approximately 90% of the products had composition claims, with no significant distinctions found across the categories. Infant cereal and cookies had a significantly higher proportion of nutrition claims (91.7% and 81.3% respectively) than did the other categories. Infant cereal also had a significantly higher proportion of health claims (85.4%) than did the other categories. No significant distinction was found across product categories for the “no added salt” claim. Only simple pureed foods and mixed foods had a “no added seasoning” claim. Infant cookies had a higher proportion of high sodium content. Infant cereal had a higher proportion of “no added sugar” claims, yet it had a higher proportion of high sugar content (68.4%), as did simple pureed foods (70.7%) (Table 2).

### Nutrition facts of different commercial CFs

Table 3 shows the nutrition facts across the food categories. Infant cookies had the highest calorie, carbohydrate, protein, fat, sugar, and sodium content of all categories. The calorie and carbohydrate contents of infant cereal were higher than those of simple pureed foods and mixed foods, but there were no significant differences in the protein content across these three product categories.

**Table 3:** Nutrition facts for different food categories ^1^.

	*n*	Calorie (kcal)	Carbohydrate (g)	Protein (g)	Total Fat (g)	Sugar ^2^ (g)	Sodium (g)	Saturated Fat (g)
**Infant cereal**	48	116.4±48.1^c^	23.3±9.6^c^	3.0±1.9^a^	1.1±0.9^a^	4.8±3.3^a^	14.0±19.0^a^	0.3±0.3^a^
**Simple puree food**	56	56.8±41.5^a^	9.9±7.3^a^	2.2±3.7^a^	1.0±2.7^a^	6.1±5.0^a^	16.0±24.1^a^	0.2±0.9^a^
**Mixed food**	211	83.4±42.3^b^	15.9±9.3^b^	2.7±1.4^a^	0.9±1.1^a^	2.4±3.1^a^	69.2±75.7^a^	0.2±0.3^a^
**Infant cookies**	48	126.2±118.1^d^	80.9±10.6^d^	6.5±3.9^b^	5.8±7.3^b^	22.1±20.0^b^	238.5±297.9^b^	2.0±3.6^b^

^1^ Data are presented as the mean ± SD of nutrient content per 100 g.

^2^ Only 237 products with sugar content data are accounted for in this table.

^a–d^ Means within each column followed by the same letter are not significantly different at the 5% level according to ANOVA with Scheffé’s test.

Table 4 lists the nutrition facts according to the age stages targeted by products. No significant differences were observed between the age stages for infant cereals. Stage 2 simple pureed foods contained more calories, protein, total fat, and sodium than did Stage 1 products. The calorie, carbohydrate, protein, fat, and sodium content of the Stage 3 mixed foods was higher than that of the Stage 1 or Stage 2 mixed foods. Stage 3 infant cookies had a significantly higher saturated fat content than did those aimed at Stages 1 and 2.

**Table 4:** Nutrition facts for different age stages ^1^.

	*n*	Calorie (kcal)	Carbohydrate (g)	Protein (g)	Total fat (g)	Sugar ^2^ (g)	Sodium (g)	Saturated fat (g)
**Infant cereal**								
** Stage 1**	37	116.8±54.5	23.3±10.9	3.0±2.1	1.2±1.0	5.3±3.2	15.5±21.0	0.3±0.3
** Stage 2**	7	122.0±12.5	24.5±1.3	3.5±1.1	1.0±0.5	5.0±3.9	11.2±10.2	0.2±0.1
***p* value**		0.173	0.144	0.163	0.911	0.804	0.772	0.629
**Simple puree food**								
** Stage 1**	52	51.5±34.3	10.6±7.1	1.4±2.3	0.5±0.7	6.3±4.9	12.6±16.2	0.1±0.2
** Stage 2**	4	124.6±70.6	1.9±3.3	12.4±3.4	7.7±7.8	0.0±0.0	60.3±58.2	1.7±3.2
***p* value**		0.007*	0.005*	0.001***	0.003**	0.214	0.005**	0.181
**Mixed food**								
** Stage 1**	78	82.3±44.8^b^	16.2±10.5^b^	2.4±1.5^a^	0.8±1.1^a^	2.3±3.5	24.1±29.6^a^	0.2±0.2
** Stage 2**	51	55.3±16.2^a^	9.9±3.2^a^	2.2±1.1^a^	0.8±0.9^a^	1.7±1.2	110.7±52.4^c^	0.2±0.3
** Stage 3**	82	102.0±41.5^c^	19.3±9.0^c^	3.2±1.3^b^	1.1±1.1^b^	3.1±3.8	86.3±94.8^b^	0.2±0.4
**Infant cookies**								
** Stage 1**	6	398.7±38.5	82.2±11.1^a^	8.6±5.5	3.8±4.4^a^^b^	8.2±7.1	532.1±300.8^b^	0.9±2.2^a^
** Stage 2**	34	397.6±35.8	82.5±10.7^b^	5.8±3.9	4.6±6.4^a^	24.8±21.4	196.8±304.2^a^	1.7±3.1^a^
** Stage 3**	8	439.6±52.3	80.9±10.6^a^	7.7±2.1	12.6±9.3^b^	26.8±3.1	195.6±101.4^a^^b^	4.4±5.3^b^

^1^ Data are presented as the mean ± SD of nutrient content per 100 g.

^2^ Only 233 products with sugar content data are accounted for in this table.

*p < 0.05;

**p < 0.01,

***p < 0.001.

^a,b,c^ Means within each column followed by the different letter are significantly different at the 5% according to Kruskal-Wallis Test.

### Calcium and iron claims versus content in infant cereal and infant cookies

Calcium content was labeled on 55 products and iron content on 33 products; 34 of the infant cereals and 21 of the infant cookies displayed calcium content, and 22 of the infant cereals and 11 of the infant cookies displayed iron content. Table 5 presents the calcium and iron content for different ages.

**Table 5:** Calcium and iron content of infant cereal or infant cookies for different age stages^1^.

	*n*	Calcium (mg)	*n*	Iron (mg)
**Infant cereal**	30	115.1±49.5	19	2.3±0.9
** Stage 1**	26	112.4±52.7	15	2.1±1.0
** Stage 2**	4	133.3±0.0	4	3.0±0.0
***p* value**		0.027*		0.099
**Infant cookies**	21	562.4±334.4	11	21.0±25.3
** Stage 1**	4	827.3±72.3	2	24.7±0.4
** Stage 2**	17	500.1±342.2	9	20.1±28.2
***p* value**		0.025*		0.097

^1^ Data are presented as the mean ± SD of nutrient content per 100 g.

*p < 0.05;

**p < 0.01,

***p < 0.001 according toMann-Whitney U test.

For calcium content, Stage 2 infant cereals had higher calcium than did Stage 1 cereals. Stage 1 infant cookies had higher calcium content than did Stage 2 cookies. No significant differences were observed in calcium content across the age stages for infant cereals or cookies.

### Food claims versus nutrition content

Table 6 shows the association between high sodium or high sugar content and food claims. High sodium content was found for 23.4% of the CFs. Approximately 20%–30% of the products with any food claim had high sodium content. In particular, the products with health claims had a significantly higher proportion of high sodium content than did those without health claims. Moreover, 5% of the products with “no added seasoning” had high sodium content.

**Table 6:** High sodium or high sugar content among products with different claims ^1^.

	*n*	High sodium (>130mg)	*p* value	*n*	High sugar (>10%)	*p* value
**Total**	363	85 (23.4)		237	129 (54.4)	
**Food claim**						
**Composition claim**						
** Yes**	320	66 (20.6)	0.001	224	122 (54.5)	0.965
** No**	43	19 (44.2)		13	7 (53.8)	
**Nutrition claim**						
** Yes**	151	34 (22.5)	0.733	131	68 (51.9)	0.386
** No**	212	51 (24.1)		106	61 (57.5)	
**Health claim**						
** Yes**	182	61 (33.5)	<0.001***	146	68 (46.6)	0.002**
** No**	181	24 (13.3)		91	61 (67.0)	
**Salt related claim**						
**No added salt**						
** Yes**	19	0 (0.0)	0.013*			
** No**	344	85 (24.7)				
**No added seasoning**						
** Yes**	78	4 (5.1)	<0.001***			
** No**	285	81 (28.4)				
**Sugar claim**						
**No added sugar**						
** Yes**				48	35 (72.9)	0.004**
** No**				189	94 (49.7)	

^1^ Data are presented as the number (percentage).

*p < 0.05;

**p < 0.01;

***p < 0.001 by chi-square test.

High sugar content was found in 54.4% of the CFs; approximately 50% of products with any food claims had high sugar content. Additionally, the products with “no added sugar” had a significantly higher proportion of high sugar content than did those without such claims.

Associations between calcium and iron content and claims were examined. Calcium content was labeled on 55 products and iron content on 33 products. The products with a “contains calcium” claim (*n* = 36; 368.8 ± 340.3 mg) had significantly more calcium than did products without such a claim (*n* = 19; 132.0 ± 87.0 mg) (*p* < 0.001). No significant difference was observed between the products with a “contains iron” claim (*n* = 22; 8.4 ± 19.9 mg) and those without such a claim (*n* = 11; 9.1 ± 8.2 mg) (*p* = 0.914).

## Discussion

### Products for different age stages

This study found that 48.2% of the commercial CFs were targeted at infants younger than 6 months, and previous studies have found that most infant food products are targeted at 4–6-month-old infants [14,16]. In Taiwan, the Ministry of Health and Welfare follows the WHO [3] recommendation that infants should be exclusively breastfed for the first 6 months before introducing them to CFs. The International Code of Marketing of Breast-Milk Substitutes, which applies to CFs for infants younger than 6 months, stipulates that CFs must not be marketed in ways that undermine exclusive and sustained breastfeeding [20]. In 2016, the World Health Assembly also asserted that inappropriate promotions for infant foods must end [21]. However, early weaning and introduction to CFs has been demonstrably influenced by parental access to commercial baby foods targeted at infants younger than 6 months [22–24]. Similar to most countries, Taiwan implemented only some aspects of the code. Only infant formula products for infants younger than 1 year are prohibited from advertising; no marketing regulations exist regarding CFs in Taiwan. Therefore, marketing regulations should be developed and implemented to curb early weaning as a result of products targeted at infants younger than 6 months.

As infants grow, they require more energy and protein from CFs. WHO complementary feeding guidelines indicated that infants aged 6–8 months require an additional 67–100 kcal per meal from CFs and infants aged 9–11 months require 75–100 kcal per meal [25]. Parents usually choose commercial CFs according to the recommended age on the food packages and may expect the products for older infants to be more nutritious. This study did not always discover an increasing trend of calorie or protein content over the age stages of the same food category. Only the calorie and protein content of Stage 3 mixed foods was higher than that of Stage 1 or Stage 2. This is consistent with a previous study that reported higher calorie and protein contents in mixed foods targeted at older infants than in those targeted at younger infants [14]. Several infant feeding guidelines recommend that complementary feeding can start with a single food, but then the variety of foods and textures should be increased according to the development of each infant [5,26]. Iron and calcium are two vital nutrients for infant health and development [1,5]. In this study, 33 products were labeled with iron content and 55 products with calcium content. Only Stage 2 products had significantly higher calcium content than those in Stage 1 products. Regulations in the United States stipulate that nutrition facts should indicate the iron and calcium content [18]; however, Taiwan does not require these two nutrients to be listed. Thus, iron and calcium content data were not available on most food packages in this study. However, infant CF packaging should be legally mandated to display the information for calcium and iron so that parents can make better choices for their infants.

### High sugar/sodium content in different food product category

Approximately 60% of the infant food was mixed food. Compared with the other categories, there was a lower percentage of mixed food with high sugar content. Several infant feeding guidelines recommend that parents should choose foods that are light in flavor and avoid foods with added sugar or sodium [1,5]. However, we found that more than 50% of the products were high in sugar. This is similar to a study conducted in the United Kingdom showing that 5.8% of commerical weaning foods had added sugar, and 10% of them had more than 10% of the total calories from sugar [16]. Moreover, more than half of infant and toddler foods in the United States were high in sugar [15]. Excessive consumption of sugar in young children is a serious public health problem, because dietary preferences develop at a young age and persist over time [27].

Several infant feeding guidelines recommend that infant cereals or simple pureed foods be the first CFs introduced to infants [5,26,28]. However, the present study found that approximately 70% of infant cereals and simple pureed foods had high sugar content, despite 50% of infant cereals having a “no added sugar” claim. This might be because some infant cereal or pure fruit puree contains natural sugar.

Moreover, a Canadian survey found that some infant cereals or baby dessert purees had more than 30% of the total calories from sugar, making them high-sugar foods [10]. Since these are usually the first CFs introduced to infants, high sugar infant cereals and high sugar puree food might lead to infants consuming excessive sugar [29]. Therefore, parents should pay careful attention to their infants’ sugar intake from infant cereals or fruit puree to avoid accustoming them to the sweet taste, to help them avoid developing chronic diseases later in life.

A previous study indicated that 83.2% of Taiwanese infants eat cookies before they are 8 months old [12]. In Taiwan, infant cookies are used as a between-meal snack or a food that can calm infants’ emotions. However, the present study found that more than 60% of infant cookies are high in sugar or sodium. These results are similar to those of other studies. In the United Kingdom, infant cookies were shown to be high in sugar [16]; in Canada, more than 30% of infant cookies had a high proportion of calories from sugar, and 12% of them contained more than 130 mg of sodium [10]. In the United States, infant cookies had an average sodium content of 486 mg, making them a high-sodium food [15]. Given these findings, parents should pay special attention to the sugar and sodium content when choosing cookies for their infants.

### High sugar/sodium content of products with different food claims

This study found that nearly 90% of products had composition claims, more than 40% had nutrition claims, and approximately half had health claims on the packaging. A previous study in Taiwan also found that 80% of infant and toddler food advertisements in parenting magazines were promoted with nutritional or health claims [13]. Such nutrition marketing is commonly used to promote infant and toddler foods, and a previous study indicated that families with young children pay particular attention to such claims [30]. Nutrition or health claims increase parents’ desire to purchase the advertised foods [8]. The present study revealed that more than 50% of products with nutrition or composition claims had high sugar content, more than 40% of products with health claims had high sugar content, and more than 30% of products with health claims had high sodium content. This finding is consistent with the finding of a previous study, which reported that 58.6% of toddler products in the United States were high in sodium and sugar, even though more than 70% of them had nutritional content claims [9]. A Canadian study also reported that 60% of child foods with such claims were high in sugar [31]. Foods with nutrition claims could mislead parents to perceive such products as more nutritious, even when they are low-nutrient foods [32]. Wong et al. [33] indicated that because of a lack of regulation in some countries, foods with nutrition claims may satisfy only one nutritional content criterion and be high in some unhealthy nutrients. A previous study found that foods with nutrition or health claims but high sugar content might lead parents to make an error in judgment and purchase such products for their children [7]. Therefore, health professionals should educate parents to understand and use the nutrition facts to consider the complete picture of food quality when selecting products, instead of simply focusing on the health or nutrition claims on food packaging.

The American Academy of Pediatrics [34] encourages choosing “infant and toddler food […] whether home or commercially prepared, with no added salt or sugar.” In this study, of 50 products with “no added sugar” claims, 35 had high sugar content. This may because the products with “no added sugar” claims had fruit ingredients. Fruits are valuable sources of fiber, vitamins, and minerals; eating fruits is a part of a healthy diet. However, García et al. [29] indicated that total sugar content in UK commercial infant food is positively correlated with fruit and vegetable content, and this might mislead parents to reinforce their children’s preference for sweet food. This study also found that of 19 products with a “no added salt” claim, 4 had high sodium content. The CAC recommends that nutrition and health claims should not be permitted for foods for toddlers younger than 3 years old, except where specifically provided for in relevant Codex standards or national legislation [11]. In the United States, nutrition claims must be accompanied with a disclosure statement if the product exceeds specified threshold levels of total fat (13 g), saturated fat (4 g), or sodium (480 mg) [35]. These policies may curb nutrition or health claims that mislead parents into selecting foods that are unsuitable for their infants’ healthy development. The results of this study indicate that pediatric health professionals must be aware of the marketing strategies commonly used on CFs, and help parents and other child caregivers to identify the true nutritional content of CFs. Future research should examine the influence of nutrition marketing on parents’ food selection.

### Limitations

This study is subject to limitations. First, the collated sample could not cover all commercial CFs in Taiwan, although we did our best to collect information on the most widely available products. Second, some products lacked sugar content information, perhaps because the labeling regulations regarding sugar content apply only to products manufactured after July 2015 [36]. Third, we did not use instrumental analysis to verify the nutritional content; thus, we could not comprehensively determine and compare the nutrient content of all sampled commercial CFs. Although we adopted nonparametric testing to account for the small sample size, the small sample size may engender a generalizability problem. Finally, complementary foods are part of a diverse diet. The use of unhealthy commercial CFs differs from unhealthy infant diets.

## Conclusions

More than 50% of Taiwanese commercial CFs have high sugar content and more than 20% were high in sodium. Moreover, products with calcium or iron content claims did not contain more calcium or iron than products without such claims. The results indicated that parents in Taiwan cannot choose the healthiest foods for their children by simply focusing on food claims. They must pay closer attention to the nutrition facts, particularly the sodium and sugar content. Pediatric physicians, dietitians, and health professionals should educate parents on how to interpret the information on food packaging so that they can choose the foods that are truly most suitable for their children’s healthy development. Given that few data for Taiwan’s commercial CFs are available, the results of this study should be useful to health professionals. Furthermore, the Taiwan government should regulate the labeling of nutrition facts and food claims for foods targeted at infants younger than 12 months.

## Supporting information

S1 File Minimal anonymized data set of food claims. (XLSX)
